# Subtle presentation of active primary biliary cirrhosis in chronic hepatitis B: a case report

**DOI:** 10.1093/gastro/gov064

**Published:** 2016-02-17

**Authors:** Asad Javaid, Mugilan Poongkunran, Felicia D. Allard, Win Kyaw, Htet Htet Maung, Daryl Lau

**Affiliations:** 1 Department of Medicine, Beth Israel Deaconess Medical Center, Harvard Medical School, Boston, MA, USA and; 2 Department of Pathology, Beth Israel Deaconess Medical Center, Harvard Medical School, Boston, MA, USA

**Keywords:** primary biliary cirrhosis, chronic hepatitis B, cholestatic disease, alkaline phosphatase, anti-mitochondrial antibodies

## Abstract

We are describing an interesting case of two chronic liver diseases in a 48-year-old Chinese woman. While chronic hepatitis B is a common entity in Asia, the patient was later found to have active, asymptomatic primary biliary cirrhosis due to a persistently elevated alkaline phosphatase level after optimal hepatitis B virus DNA suppression on antiviral therapy. This report emphasizes the importance of keeping a high index of suspicion for another potential liver disease process even after a patient has been successfully treated for a primary liver condition. Clinical vigilance, especially in atypical clinical presentations, can result in early accurate diagnosis and prompt treatment.

## Introduction

Hepatitis B virus (HBV) infection is a global public health problem affecting >400 million people worldwide. It is estimated that there are 1.2 million deaths per year due to complications such as liver failure and hepatocellular carcinoma (HCC) [1]. The prevalence of chronic hepatitis B varies in different parts of the world [2]. The highest rates of hepatitis B are found in continents such as Asia and Africa, where perinatal or horizontal transmissions of HBV at infancy and early childhood are common [1].

While HBV infection is common among Asians, primary biliary cirrhosis (PBC) is considerably less common. PBC is a chronic cholestatic liver disease with autoimmune etiology. It is characterized by progressive destruction of small intrahepatic bile ducts and portal inflammation leading to fibrosis and eventually to cirrhosis [3]. It primarily affects women, with a peak incidence in the fifth decade of life, and is more prevalent in developed nations. In fact, the highest annual incidence (2.7/100 000 per year) and prevalence (40.2/100000 per year) for PBC are found in the USA [4].

To date, there are very few reports on the concurrent diagnosis of chronic hepatitis B and PBC among Asians. Here, we report a unique case of a 48-year-old Chinese woman with both PBC and HBV. Our report highlights the importance of considering less common conditions in the differential diagnosis when the clinical presentation is atypical.

## Case Presentation

Our patient is a 48-year-old woman who immigrated to the United States from China in 2010. She was diagnosed with hepatitis B e antigen (HBeAg) positive chronic hepatitis B in the early 1990s at a health-screening program in China. Her past medical history is otherwise insignificant. She worked as an electrician in China. Her major risk factor for hepatitis B was living in an endemic area for >40 years. Her son also has hepatitis B. The HBV status of her parents and three brothers are unknown. Her father died of lung cancer, and her mother died of heart disease at the age of 40 years. There is no family history of autoimmune conditions. In 2008, she was noted to have active hepatitis and was started on Entecavir (ETV) 0.5 mg daily. A baseline liver biopsy was also performed in China, but the pathology report was not available. She was compliant in taking antiviral therapy until she relocated to the USA.

**Figure 1.**
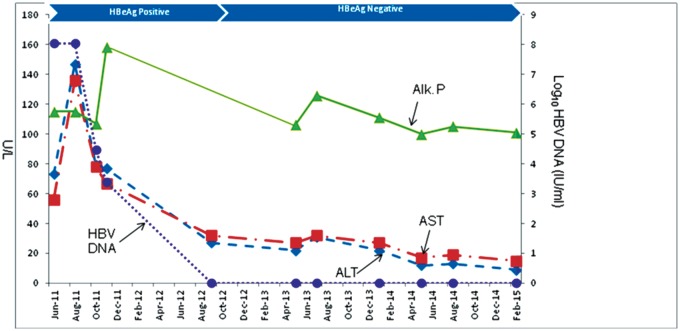
Clinical course after start of antiviral therapy for chronic hepatitis B in August 2011. The patient achieved undetectable HBV DNA and HBeAg seroconversion in September 2012. UDCA was started in February 2014 for PBC, with normalization of ALP.

Initial investigation at our liver center in June 2011 showed elevated serum alanine aminotransferase (ALT) and aspartate aminotransferase (AST) at 73 and 56 U/L, respectively. The HBV DNA was >110 million IU/mL. HBeAg was reactive, while anti-HBe was negative. Her hepatic synthetic function, alpha-fetoprotein, renal function and abdominal ultrasound were all within normal limits. By August 2011, her ALT and AST had increased to 147 and 136 IU/L, respectively, with HBV DNA at >110 million IU/mL. ETV 0.5 mg daily was restarted. She had rapid virological and biochemical responses to therapy and achieved HBeAg seroconversion by September 2012 (Figure 1). While ALT and AST levels returned to normal on therapy, her serum alkaline phosphatase (ALP) levels continued to be elevated >105 U/L (the upper limit of normal). She had no clinical, laboratory or ultrasound features of advanced hepatic fibrosis. Her serum fibrosis panel (Hepascore) was 0.19, which is consistent with stage 0 hepatic fibrosis.

**Figure 2.**
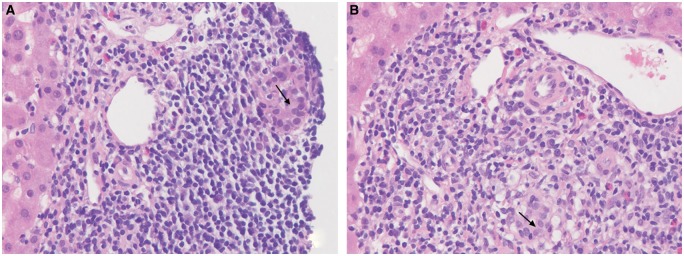
High-power (400×) photomicrographs demonstrate interlobular bile ducts infiltrated by lymphocytes (lymphocytic cholangitis) with associated epithelial damage. The bile ducts are surrounded by a dense aggregate of inflammatory cells comprising predominantly lymphocytes with admixed plasma cells and macrophages (macrophages best demonstrated in **panel B**). These findings are diagnostic of a non-suppurative destructive cholangitis consistent with early (stage 1) PBC. The arrow denotes the bile duct lumen.

**Figure 3.**
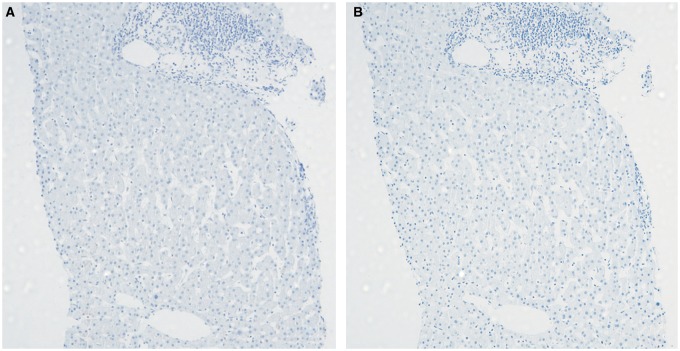
**Panel A** demonstrates negative HBsAg staining, while **panel B** demonstrates negative hepatitis B core antigen (40× magnification).

Further evaluation reported positive anti-mitochondrial antibodies (AMA) at a titer of 1:640. The antinuclear antibody and anti-smooth muscle antibody were repeatedly negative. Subsequent liver biopsy revealed moderate-to-severe portal inflammation (predominantly comprising lymphocytes) and occasional apoptotic hepatocytes. In addition, there were foci of marked bile duct injury with infiltrating lymphocytes and focal bile ductular proliferation (Figure 2). Trichrome staining demonstrated portal tracts expanded by inflammatory infiltrate, areas of increased portal fibrosis and focal early periportal fibrosis (stage 1 fibrosis). There was no steatosis or stainable iron. Immunostains for HBsAg and HBcAg were negative (Figure 3). The pathology findings of non-suppurative destructive cholangitis were consistent with early PBC. The patient had no history of autoimmune diseases, pruritus or extrahepatic manifestations of PBC. She was subsequently started on ursodeoxy-cholic acid (UDCA) at 15 mg/kg. Within a month on UDCA, her ALP decreased to 100 U/L. She was last monitored in the liver clinic on 26 August 2015. She remained asymptomatic, and her liver condition was stable with normal ALT (9 U/L), AST (15 U/L), ALP (98 U/L) and undetectable HBV DNA.

## Discussion

We describe a case of chronic hepatitis B co-existing with PBC in a 48-year-old Asian woman. While hepatitis B had been diagnosed in her 20 s, PBC was silent and was only diagnosed in her 40 s due to persistently mild elevation of ALP after optimal control of HBV. The subsequent liver biopsy reported characteristic features of intrahepatic ductal injury and portal inflammation.

PBC is a slowly progressive autoimmune disease that mainly affects women [5]. It was believed to be more prevalent in northern Europe and northern America compared with eastern Asia, Africa and Australia [6]. A recent study from southern China reported a prevalence of 49.2 cases of PBC per 100000 adults who presented for routine annual examinations [7]. This result is actually similar to the prevalence stated in other geographical locations. The higher reported rates of PBC could be secondary to improved diagnostic laboratory abilities with automated equipment and increased physician awareness of the disease [7].

Compared with other causes of chronic liver diseases in Asian populations, PBC remains uncommon. Large cohort studies in Japan and Hong Kong have reported only 2.4% and 1.3% of PBC cases among all of their patients with chronic liver disease, respectively. More than 90% of the PBC cases were identified in women [8,9]. The concurrent existence of PBC and HBV is rare. In a recently published study in China, Bo-An Li *et al*. reported presence of the AMA-M2 antibody in 22 of 325 (6.8%) patients with chronic hepatitis B [10].

The diagnosis of PBC in patients with chronic hepatitis B is usually delayed by many years in the majority of reported cases [11]. The reason for the early diagnosis of HBV, especially in the endemic regions of the world, can be attributed to a high degree of hepatitis B awareness and availability of simple serological tests. Furthermore, the majority of patients with PBC have no symptoms, similar to our case. The relatively higher prevalence of PBC noted by Li *et al.* could be attributed to increased disease awareness, availability of diagnostic tests or a changing epidemiology of PBC [10].

The prevalence of PBC in Asian patients who attended a Hong Kong hepatology clinic in 2004 was 1.3%, while the prevalence of chronic hepatitis B in the same time period was 89.4%. The authors noted a lower rate (2.5%) of HBV infection among PBC patients compared with about 8.8% in their general population [9]. Similarly, Taiwan has a HBV prevalence of 15%, but only 1 of 26 (3.8%) PBC patients tested positive for HBsAg [12]. In a case control study conducted in Taiwan, the authors observed that none of the 27 PBC patients enrolled in the study were HBsAg sero-reactive, while 17 of 108 (15.7%) of the age- and sex-matched healthy controls were HBsAg positive (*P* = 0.022) [13]. None of these studies suggested that HBV affects the disease progression of PBC [9,12,13]. Due to the relatively small number of PBC patients in these studies, one could not assume that PBC has a protective role in HBV transmission.

In a retrospective study conducted in Greece, 9 of 1483 (0.6%) patients with HBV had concurrent PBC (11). In that study population, one of the most common presenting symptoms was pruritus, and some patients experienced severe pruritus many years before being diagnosed. In seven of nine patients, PBC was diagnosed 106 (± 89) months after the diagnosis of HBV [11]. This delay in diagnosis might be due to a low index of suspicion.

Approximately 50–60% of patients with PBC are asymptomatic at diagnosis [5]. Among the symptomatic patients, pruritus is the most common presenting symptom and occurs in about 50% of patients [14]. A suspicion of PBC is often raised based on biochemical evidence of cholestasis with elevation of serum ALP. High concentrations of ALP can be found in liver and bone, followed by kidney, intestine and placenta [15]. Chronic cholestasis and infiltrative liver disease should be suspected if the ALP elevation is persistent and of liver origin [16]. Common differential diagnoses of cholestatic liver diseases include anabolic steroid-induced cholestasis, PBC, primary sclerosing cholangitis and adult bile ductopenia [16]. Major infiltrative diseases are sarcoidosis, other granulomatous diseases or metastatic cancer to liver [16].

AMA is considered to be the serological hallmark of disease as it is present in 90–95% of the affected population. AMA may be elevated in other medical conditions, but the E2 subtype is believed to be specific to PBC [5,14]. Antinuclear antibody is detectable in approximately 50% of patients with PBC and can lead to the misdiagnosis of autoimmune hepatitis.

Liver biopsy is invaluable for making a diagnosis of co-existing liver diseases. The diagnosis of PBC may be straightforward if characteristic histologic findings such as florid duct lesions, mononuclear-cell-predominant portal inflammation and duct-infiltrating lymphocytes are present, as they were in this case. Some reports suggest that these classical findings are apparent only in the early stage of PBC. In its advanced stage, it may be more difficult to differentiate PBC from other chronic liver diseases [17]. UDCA is an effective therapy for PBC that suppresses the release of endogenous bile acids, which are toxic to hepatocytes, and inhibits eosinophil activation [18]. UDCA is the first-line therapy for PBC and has been shown to reduce mortality [19]. Our patient’s response to UDCA was evident by the normalization of her ALP.

This case emphasizes that a patient can have more than one liver disease. Any atypical clinical presentation or treatment response pattern should raise the possibility of another concurrent liver condition. In this case, the persistent elevation of ALP provided a clue to the diagnosis of PBC in an otherwise well-controlled hepatitis B patient. This case also illustrated the importance of a detailed, systematic work-up to identify co-existing liver disease and allow early initiation of therapy for a better prognosis.

*Conflict of interest statement*: none declared.
